# Spatial accuracy of computed tomography perfusion to estimate the follow-up infarct on diffusion-weighted imaging after successful mechanical thrombectomy

**DOI:** 10.1186/s12883-023-03075-z

**Published:** 2023-01-20

**Authors:** Xiao-Quan Xu, Gao Ma, Guang-Chen Shen, Shan-Shan Lu, Hai-Bin Shi, Ya-Xi Zhang, Yu Zhang, Fei-Yun Wu, Sheng Liu

**Affiliations:** 1 Department of Radiology, The First Affiliated Hospital of Nanjing Medical University, Nanjing, China; 2 Department of Interventional Radiology, The First Affiliated Hospital of Nanjing Medical University, Nanjing, China; 3 Shukun Network Technology Co., Ltd, Beijing, China

**Keywords:** Stroke, Thrombectomy, Computed tomography perfusion, Infarct, Location

## Abstract

**Background:**

Volumetric accuracy of using computed tomography perfusion (CTP) to estimate the post-treatment infarct in stroke patients with successful recanalization after mechanical thrombectomy (MT) has been studied a lot, however the spatial accuracy and its influence factors has not been fully investigated.

**Methods:**

This retrospective study reviewed the data from consecutive anterior large vessel occlusion (LVO) patients who had baseline CTP, successful recanalization after MT, and post-treatment diffusion-weighed imaging (DWI). Ischemic core on baseline CTP was estimated using relative cerebral blood flood (CBF) of < 30%. The infarct area was outlined manually on post-treatment DWI, and registered to CTP. Spatial agreement was assessed using the Dice similarity coefficient (DSC) and average Hausdorff distance. According to the median DSC, the study population was dichotomized into high and low Dice groups. Univariable and multivariable regression analyses were used to determine the factors independently associated with the spatial agreement.

**Results:**

In 72 included patients, the median DSC was 0.26, and the median average Hausdorff distance was 1.77 mm. High Dice group showed significantly higher median ischemic core volume on baseline CTP (33.90 mL vs 3.40 mL, *P* < 0.001), lower proportion of moderate or severe leukoaraiosis [27.78% vs 52.78%, *P* = 0.031], and higher median infarct volume on follow-up DWI (51.17 mL vs 9.42 mL, *P* < 0.001) than low Dice group. Ischemic core volume on baseline CTP was found to be independently associated with the spatial agreement (OR, 1.092; P < 0.001).

**Conclusions:**

CTP could help to spatially locate the post-treatment infarct in anterior LVO patients who achieving successful recanalization after MT. Ischemic core volume on baseline CTP was independently associated with the spatial agreement.

## Introduction

Stroke is the leading cause of disability and mortality in the worldwide [1]. The efficacy of mechanical thrombectomy (MT) has been well proven up to 24 hours after symptom onset in patients with acute ischemic stroke (AIS) due to anterior large vessel occlusion (LVO) if selected using advanced imaging [2, 3]. Precise assessment of the “tissue window” on baseline imaging is very important for identifying the patients eligible for MT in extended time window [4, 5]. Currently, computed tomography perfusion (CTP) is widely used to estimate the volume of the ischemic core and hypoperfusion areas, and then to identify the status of “tissue window” in stroke patients with a onset time between 6 and 24 hours [6, 7]. However, the volumetric and spatial accuracy of CTP estimated ischemic core in the current era of MT are still uncertain up to now.

Previously, some studies have tried to explore the volumetric agreement between the ischemic core estimated on CTP and the final infarct on post-treatment magnetic resonance imaging (MRI) after successful MT [8–10]. Despites the potential ischemic core overestimation on CTP in stroke patients with poor collateral status and shorter time interval from symptom onset to imaging [11], multiple studies have reported the high agreement of infart volume between initial CTP and follow-up diffusion weighted imaging (DWI) or fluid attenuation inversion recovery (FLAIR) imaging, especially in early and fully recanalized patients [9, 12–14]. However, few studies have assessed the accuracy, especially its influence factors of using CTP to spatially locate the post-treatment infarct in stroke patients after successful MT until now. Accurate spatial estimation of infarct area is of great importance due to its close association with the clinical outcomes of stroke patients, and its subsequent impact on the eligibility for MT procedure [9, 15].

Therefore, the purpose of this study was to evaluate the accuracy and its influence factors of using CTP to spatially locate the post-treatment infarct in stroke patients with successful recanalization after MT.

## Materials and methods

### Patients

This was a retrospective cohort study of patients with AIS who underwent MT between October 2019 and December 2021. The study protocol was approved by the Ethics Committee of the First Afliated Hospital of Nanjing Medical University. The ethics committee waived the written informed consent because of that present study was retrospective. The inclusion criteria were as follows: 1) diagnosis of AIS; 2) anterior circulation occlusion (internal carotid artery and/or middle cerebral artery); 3) baseline CTP and follow-up DWI were performed; 4) successful recanalization was achieved after MT; 5) the imaging quality was adequate for subsequent analyses. We excluded the patients with a ischemic core volume of 0 mL on baseline CTP. Successful recanalization was defined as a modified Treatment in Cerebral Infarction (mTICI) score ≥ 2b [16]. Besides that, sensitivity analysis was performed in patients achieving mTICI 2c or 3.

### Clinical data

We collected the following demographic and clinical information from the electronic medical records: gender, age, stroke related risk factors (hypertension, hyperlipidemia, diabetes, atrial fibrillation, smoker), baseline National Institutes of Health Stroke Scale (NIHSS) score, intravenous thrombolysis was performed or not, the time interval from stroke onset to CTP, the time interval from CTP to recanalization, and the modified Rankin Scale (mRS) score at 3 months after treatment. The clinical outcome was assessed based on the mRS score which was a standardized 6-point score that measures the functional independency of patients with acute stroke at 90 days after treatment. Good functional outcome was defined as a mRS ≤ 2 [17].

### Baseline imaging protocol

All the patients with AIS underwent baseline CT scan on a 128-section CT scanner (Optima CT 660; GE Healthcare), including noncontrast CT (120 kV, 100–350 auto-mAs, contiguous 5-mm axial sections) and whole-brain volumetric CTP (four-dimensional adaptive spiral mode, periodic spiral approach, 80 mm in z-axis, 2-second delay after the start of contrast medium injection, temporal resolution of 1.7 sec, total CTP duration of 53 sec, 100 kVp, 200 mAs, rotation time of 0.4 sec, 0.984 maximum pitch). CTP acquisition was started after initiation of intravenous injection of 50 mL of iopromide (Iopromide, Ultravist 370, Bayer Schering Pharma) at a rate of 5 mL/sec. Contrast was followed by a 30 mL saline flush at the same rate. Simulated CT angiography (CTA) images (section thickness, 0.625 mm) were reconstructed from the peak arterial phase of CTP source images.

### Post-treatment MRI protocol

Follow-up MRI scan was performed with a 3 T scanner (Magnetom Skyra, Siemens Healthcare) equipped with a 20-channel head-neck coil, usually within 7 days after treatment. The MRI protocol included DWI, FLAIR, susceptibility-weighted imaging (SWI), MR angiography (MRA) and arterial spin labeling (ASL). DWI were scanned using the following parameters: repetition time (TR) = 6400 msec; echo time = 98 msec; b value = 0 and 1000 mm^2^/s; field-of-view (FOV) = 220 × 220 mm^2^; matrix = 192 × 192; section thickness = 4 mm; number of slices = 20. The time interval from CTP scan to follow-up MRI scan was also recorded.

### Imaging analyses

The location of intracranial LVO was evaluated based on the simulated CTA, was defined as intracranial ICA or not [16]. CTP data was post-processed with a commercial software named CTPdoc AI (SHUKUN, Beijing, China), by using the oscillation index singular value decomposition (oSVD) deconvolution method which was widely applied in congeneric automated perfusion analysis softwares (e.g., RAPID) [18]. Predefined relative CBF threshold value of < 30% at CTP was used to calculate the volume of baseline ischemic core [18]. We assessed the occurrence and degree of leukoaraiosis based on the pre-treatment CT images according to the method proposed in previous study [19]. The degree of leukoaraiosis was dichotomized into absent or mild (Fazekas scores, 0–1) versus moderate or severe (Fazekas scores, 2–3) [19]. The volume of cerebral infarct was measured by a neuro-radiologist (with 10 years of experience), based on the follow-up DWI. After the bordline of infarct areas was manually outlined section by section based on the ITK-SNAP software (version 3.8, http://www.itksnap.org), the volume could be aumatically obtained. Hemorrhage transformation (HT) was assessed based on the post-treatment CT. If uncertain, SWI was referred. We included the HT area that was located within the infarct area in the delineation. The area of infarct on each section could be obtained after manual delineation. Then, the volume of infarct on follow-up DWI could be calculated by multiplying the sum of infarction areas by the slice thickness. The registration between CTP and DWI images is done by an automated image analysis toolkit (SHUKUN, Beijing, China), which is based on a Python library named ANTsPy [20]. CTP and DWI lesion overlap was calculated using SciPy [21] and spatial agreement assessed using SciPy and scikit-image [22]. The Dice similarity coefficient was calculated to assess spatial agreement between CTP and DWI lesions [20]. We also used the average Hausdorff distance (the average of all minimum distances between two segmentations) to quantify the spatial agreement [23]. The average Hausdorff distance was calculated using Formula 2 given by Aydin OU et al. [24].

### Statistical analysis

Normality test of quantitative variables were performed using the Shapiro-Wilk test. If normally distributed, the variables were expressed as mean ± standard deviation (SD). If not, they were reported as median with interquartile range (IQR). The categorical variables were reported as numbers and proportions. According to the median Dice similarity coefficient, we dichotomized the study population into high and low Dice groups. According to the median Hausdorff distance, we also dichotomized the study population into high and low Hausdorff distance groups. For categorical variables, Chi-square tests were used to compare the differences. For quantitative variables in normal distribution, independent sample t tests were used to compared the differences. For those not in normal distribution, Mann-Whitney U tests were used to compare the differences. Spearman correlation analyses were applied to assess the correlations between the Dice similarity coefficient and the significantly different variables. The variables with *P* values less than 0.1 in univariable analysis were included into the multivariable logistic regression to assess the independent factor associated with the Dice similarity coefficient. Bland-Altman plots were used to illustrate mean differences and limit of agreement (LoA), and the intraclass correlation coefficient (ICC) was used to assess the agreement between the volume of ischemic core on baseline CTP and the volume of infarct area on follow-up DWI. All statistical analyses were performed using SPSS software (version 23.0) and MedCalc software (version 20.1). Two-sided *P* value less than 0.05 was considered statistically significant.

## Results

According to the inclusion criteria, 72 patients were finally included. Patients characteristics are detailed in Table 1. Of the included 72 patients, 49 (68.06%) patients were men. The median age was 69 (62–74) years. Hypertension, hyperlipidemia, diabetes, atrial fibrillation and current smoker were found in 51 (70.83%), 3 (4.17%), 14 (19.44%), 22 (30.56%) and 20 (27.78%) patients, respectively. The mean baseline NIHSS score 14 ± 7. The median time interval from symptom onset to CTP scan was 279.50 minutes (IQR, 190.00–384.50 minutes). The median time interval from CTP to recanalization was 46.00 minutes (IQR, 38.00–62.00 minutes). 25 (34.72%) patients had a intracranial ICA occlusion. Moderate or severe leukoaraiosis was found in 29 (40.28%) patients. The median volume of ischemic core on baseline CTP was 11 (3–35) mL. The median volume of infarct area on follow-up DWI was 23 (7–59) mL. The median time interval from baseline CTP to follow-up MRI scan was 4 (IQR, 3–5; range, 1–7) days. The median Dice similarity coefficient was 0.26 (0.14–0.44), and the median average Hausdorff distance was 1.77 (0.31–4.48) mm. 25 (34.72%) patients were treated with intravenous thrombolysis before MT. After treatment, HT was observed in 16 (22.22%) patients, and good outcome was achieved in 55 (76.39%) patients.

**Table 1:** Baseline patient characteristics

Variables	Numbers
Median age, y (IQR)	69 (62–74)
Sex, n(%) male	49 (68.06%)
Hypertension, n (%)	51 (70.83%)
Hyperlipidemia, n (%)	3 (4.17%)
Diabetes, n (%)	14 (19.44%)
Smoker, n (%)	20 (27.78%)
Atrial fibrillation, n (%)	22 (30.56%)
Mean baseline NIHSS, (SD)	14 ± 7
Intravenous thrombolysis, n (%)	25 (34.72%)
Median onset to CTP time, min (IQR)	279.50 (190.00–384.50)
Median CTP to recanalization time, min (IQR)	46.00 (38.00–62.00)
Intracranial ICA occlusion, n (%)	25 (34.72%)
Median ischemic core volume on baseline CTP, mL (IQR)	11 (3–35)
Moderate or severe leukoaraiosis, n (%)	29 (40.28%)
Median infarct volume on follow-up DWI, mL (IQR)	23 (7–59)
Median Dice similarity coefficient (IQR)	0.26 (0.14–0.44)
Median average Hausdorff distance, mm (IQR)	1.77 (0.31–4.48)
Hemorrhage transformation, n (%)	16 (22.22%)
Good outcome, n (%)	55 (76.39%)

IQR indicates inter-quartile range; NIHSS, National Institutes of Health Stroke Scale scores; SD, standard deviation; CTP, computed tomography perfusion; ICA, internal carotid artery; DWI, diffusion-weighted imaging

Categorical variables were expressed as absolute numbers (percentages)

Continuous variables were displayed as mean ± SD if normally distributed, or median (IQR) if not normally distributed

As to the volumetric analysis, Bland-Altman analyses indicated that the mean difference between the volume of ischemic core on baseline CTP and the volume of infarct area on follow-up DWI was 13.0 mL (LoA, 72.7 to − 46.7). The ICC between two volumetric parameters was 0.68 (95%CI, 0.46–0.81). There were 6 patients with 5 to 10 mL core overestimation, and 9 patients with > 10 mL core overestimation.

**Fig. 1:**
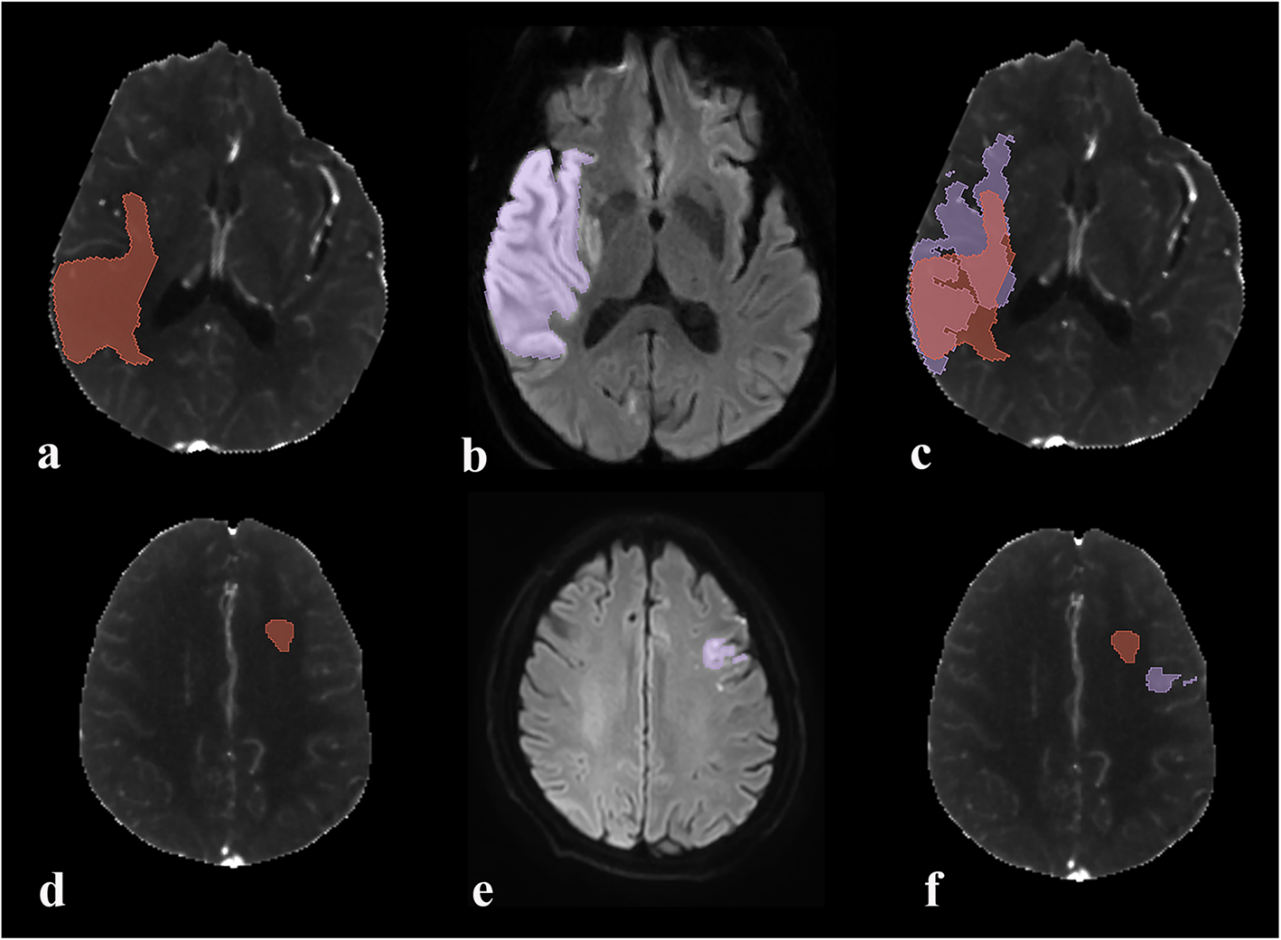
Assessment of spatiall agreement in two representative patients. **a** - **c** One 77-year-old male sufferred from the occlusion of right middle cerebral artery. The time interval from symptom onset to CTP scan was 324 minutes, and the time interval from CTP to recanalization was50 minutes. The volume of ischemic core on baseline CTP and infarct area on follow-up DWI was 81.1 mL and 118.7 mL, respectively. After the ischemic core was identified on CTP- derived CBF maps **a** and infart area was manually outlined on post-treatment DWI **b**, infarct area on post-treatment DWI was registered to CTP **c**. The Dice similarity coefficient was 0.70, and the average Hausdorff distance was 0.35 mm. **d** - **f** One 47-year-old male sufferred from the occlusion of left internal carotid artery. The time interval from symptom onset to CTP scan was 199 minutes, and the time interval from CTP to recanalization was 85 minutes. The volume of ischemic core on baseline CTP was 2.60 mL, and the volume of infarct area on follow-up DWI was 2.23 mL. After the ischemic core was identified on CTP- derived CBF maps **d** and infart area was manually outlined on post-treatment DWI **e**, infarct area on post-treatment DWI was registered to CTP **f**. The Dice similarity coefficient was less than 0.01, and the average Hausdorff distance was 16.85 mm

According to the median Dice similarity coefficient, the study population was dichotomized into high (*n* = 36) and low Dice group (n = 36). As a result, we found that, high Dice group showed significantly higher median ischemic core volume on baseline CTP (34 [IQR, 17–47] mL vs 3 [IQR, 2–10] mL, *P* < 0.001), lower proportion of moderate or severe leukoaraiosis [27.78% vs 52.78%, *P* = 0.031], and higher median infarct volume on follow-up DWI (51 [IQR, 19–78] mL vs 9 [IQR, 5–24] mL, P < 0.001) than low Dice group. Besides that, compared with the low Dice group, although relatively shorter median time interval from symptom onset to CTP scan (233.00 [IQR, 177.50–333.50] vs 304.00 [IQR, 214.50–440.00], *P* = 0.067), and shorter median time interval from CTP to recanalization (44.50 [IQR, 33.50–61.00] vs 48.00 [IQR, 41.00–77.50], *P* = 0.092) were observed in the high Dice group, however the differences did not reach significant. As to the other variables, there were no significant differences between high and low Dice group (all *P* > 0.05) (Table 2). Assessments of the spatiall agreements in two representative patients were shown in the Fig. 1.

**Table 2:** Comparison of variables between high and low Dice groups

Variables	High Dice group (n = 36)	Low Dice group (***n*** = 36)	***P*** value
Median ischemic core volume on baseline CTP, mL (IQR)	34 (17–47)	3 (2–10)	< 0.001
Median infarct volume on follow-up DWI, mL (IQR)	51 (19–78)	9 (5–24)	< 0.001
Moderate or severe leukoaraiosis, n (%)	10 (27.78%)	19 (52.78%)	0.031
Median onset to CTP time, min (IQR)	233.00 (177.50–333.50)	304.00 (214.50–440.00)	0.067
Median CTP to recanalization time, min (IQR)	44.50 (33.50–61.00)	48.00 (41.00–77.50)	0.092
Mean baseline NIHSS, (SD)	16 ± 8	13 ± 6	0.148
Hypertension, n (%)	23 (63.89%)	28 (77.78%)	0.195
Intravenous thrombolysis, n (%)	15 (41.67%)	10 (27.78%)	0.216
Intracranial ICA occlusion, n (%)	15 (41.67%)	10 (27.78%)	0.216
Hemorrhage transformation, n (%)	10 (27.78%)	6 (16.67%)	0.257
Sex, n(%) male	26 (72.22%)	23 (63.89%)	0.448
Median age, y (IQR)	67 (60–75)	69 (63–73)	0.844
Hyperlipidemia, n (%)	1 (2.78%)	2 (5.56%)	> 0.999
Diabetes, n (%)	7 (19.44%)	7 (19.44%)	> 0.999
Smoker, n (%)	10 (27.78%)	10 (27.78%)	> 0.999
Atrial fibrillation, n (%)	11 (30.55%)	11(30.55%)	> 0.999

IQR indicates inter-quartile range; NIHSS, National Institutes of Health Stroke Scale scores; SD, standard deviation; CTP, computed tomography perfusion; ICA, internal carotid artery; DWI, diffusion-weighted imaging

Categorical variables were expressed as absolute numbers (percentages)

Continuous variables were displayed as mean ± SD if normally distributed, or median (IQR) if not normally distributed

Spearman correlation analyses indicated that, the median Dice similarity coefficient correlated significantly with the median ischemic core volume on baseline CTP (r = 0.699, *P* < 0.001), the degree of leukoaraiosis (r = − 0.235, *P* = 0.047), and the median infarct volume on follow-up DWI (r = 0.681, *P* < 0.001).

According to the predefined threshold value (*P* = 0.1), the following five variables (ischemic core volume on baseline CTP, moderate or severe leukoaraiosis, infarct volume on follow-up DWI, time interval from symptom onset to CTP scan, time interval from CTP to recanalization) were adopted into multivariable logistic regression analysis. As a result, we found that the ischemic core volume on baseline CTP was independently associated with the median Dice similarity coefficient (OR, 1.092; 95%CI, 1.046–1.140; *P* < 0.001).

Sensitivity analysis was performed in patients achieving mTICI 2c or 3. As a results, we found that there was no significant difference on Dice similarity coefficient between mTICI 2c/3 and mTICI 2b group (0.33 [95%CI, 0.16–0.32; IQR, 0.15–0.43] vs 0.24 [95% CI, 0.23–0.38; IQR, 0.15–0.43], *P* = 0.445).

According to the median Hausdorff distance, the study population was also dichotomized into high (*n* = 36) and low Hausdorff distance group (n = 36). As a result, we found that, high Hausdorff distance group showed significantly lower median infarct volume on follow-up DWI (11 [IQR, 5–25] mL vs 38 [IQR, 16–78] mL, *P* < 0.001) than low Hausdorff distance group. As to the other variables, there were no significant differences between high and low Hausdorff distance group (all *P* > 0.05) (Table 3).

**Table 3:** Comparison of variables between high and low Hausdorff distance groups

Variables	High Hd group (n = 36)	Low Hd group (n = 36)	***P*** value
Median ischemic core volume on baseline CTP, mL (IQR)	10(3–33)	19(3–36)	0.888
Median infarct volume on follow-up DWI, mL (IQR)	11(5–26)	38(16–78)	< 0.001
Moderate or severe leukoaraiosis, n (%)	18(50.00%)	11(30.55%)	0.093
Median onset to CTP time, min (IQR)	223.50(162.00–362.50)	287.00(226.00–523.50)	0.044
Median CTP to recanalization time, min (IQR)	48.500(38.500–80.500)	44.50(38.00–60.50)	0.228
Mean baseline NIHSS, (SD)	15 ± 7	14 ± 7	0.516
Hypertension, n (%)	29(80.56%)	22(61.11%)	0.070
Intravenous thrombolysis, n (%)	12(33.33%)	13(36.11%)	0.804
Intracranial ICA occlusion, n (%)	11(30.55%)	14(38.89)	0.458
Hemorrhage transformation, n (%)	5(13.89%)	11(30.55%)	0.089
Sex, n(%) male	23(63.89%)	26(72.22%)	0.448
Median age, y (IQR)	68(63–75)	69(57–72)	0.300
Hyperlipidemia, n (%)	2(5.56%)	1(2.78%)	> 0.999
Diabetes, n (%)	5(13.89%)	9(25.00%)	0.176
Smoker, n (%)	7(19.44%)	13(36.11%)	0.114
Atrial fibrillation, n (%)	10 (27.78%)	12(33.33%)	0.609

Hd indicates Hausdorff distance; IQR, inter-quartile range; NIHSS, National Institutes of Health Stroke Scale scores; SD, standard deviation; CTP, computed tomography perfusion; ICA, internal carotid artery; DWI, diffusion-weighted imaging

Categorical variables were expressed as absolute numbers (percentages)

Continuous variables were displayed as mean ± SD if normally distributed, or median (IQR) if not normally distributed

## Discussion

Our study had three main findings. First, if using the baseline CTP to spatially locate the post-treatment infarct in stroke patients after MT, the median Dice similarity coefficient was about 0.26, and the median average Hausdorff distance was about 1.77 mm based on our study population. Second, the high Dice group showed significantly higher median ischemic core volume on baseline CTP, lower proportion of moderate or severe leukoaraiosis, and higher median infarct volume on follow-up DWI than low Dice group. Third, the ischemic core volume on baseline CTP was found to be an independent factor associated with the spatial agreement. Thereby, our results might help the physicians to estimate the spatial location of the post-treatment infarct based on the baseline CTP in stroke patients with successful recanalization after MT, and then to establish a reasonable treatment strategy for the stroke patients.

In our study, the median Dice similarity coefficient was about 0.26 when using the baseline CTP to spatially locate the post-treatment infarct in stroke patients after MT. This result was similar with the results of two previous studies of Hoving JW et al. [8, 10]. Based on a study cohort of 120 patients with successful endovascular reperfusion, they reported a median Dice similarity coefficient of 0.24 when also using a relative CBF of < 30% to define the ischemic core [10]. Subsequently, they applied four different approaches for ischemic core estimation (cerebral blood volume < 1.2 mL/100 mL with and without smoothing filter, relative CBF < 30%, and relative CBF < 20%). As a result, they found that the median Dice similarity coefficients were ranged from 0.16 to 0.21 [8]. Although the DICE similarity coefficient in our study was to be close to those reported in the studies of Hoving JW et al. [8, 10], we should report that the time points of follow-up MRI were different. Follow-up imaging was performed at median 24.4 hours in their study [8], while 4 days in our study. The longer time interval and the potential infarction growth might result in a realtively higher DICE similarity coefficient in our study. Besides that, both two groups studies the potential influence of mTICI on the Dice similarity coefficient. Although the proportion of mTICI 2c/3 was different between our study and the study of Hoving JW et al. (48/72 vs 41/120), both two groups found that the mTICI might have no obvious influence on the Dice similarity coefficient.

However, our results were different from that of Rava RA et al. [9]. They reported the Dice similarity coefficients were 0.60 ± 0.03, 0.60 ± 0.02 and 0.44 ± 0.08, when Sphere, Vitrea and RAPID softwares were used to automatically estimate the ischemic core, respectively [9]. In my opinion, the differences among our studies were associated with the differences of patients characteristics, particularly the ischemic volume. The median volume of ischemic core on baseline CTP was 11.05 mL in our study, and 7.8 mL in the study of Hoving JW et al. [10], while it was more than 50 mL in the study of Rava RA et al. [9]. Compared with the large-sized ischemic area, the difficulty in spatially locating the ischemic area with limited extent would greatly increase. Simultaneously considering that the ischemic core volume on baseline CTP was found to be an independent factor associated with the spatial agreement, it was not surprising that the median Dice similarity coefficients of our study and the studies of Hoving JW et al. would be lower than that of Rava RA et al. [9, 10].

Originally, we expected that the low Dice group would show a significantly longer median time interval from CTP to recanalization than high Dice group. Longer time interval would lead to more infarct growth, subsequently result in decreased spatial agreement. However, in our study, we did not find a significant difference on the time interval from CTP to recanalization between high and low Dice group. In my opinion, it might be due to that recanalization did not always mean the reperfusion [23]. Despite upstream larger artery was recanalized, the downstream microvessel might be still obstructed due to the pericyte contraction, endothelial cell swelling, and luminal clogging with leukocytes and microthrombi [23, 25]. Despite fast recanalization was achieved after CTP scan, microvascular dysfuncation might lead to the continuous impaired tissue perfusion and infarct growth, and subsequently result in the no-relationship between the spatial agreement and the time interval from CTP to recanalization.

Leukoaraiosis was a common radiologic sign indicating the age-related white matter damage in the penetrating small vessel territory [19]. Previously, Rudilosso S et al. found that leukoaraiosis might confound the interpretation of CTP in stroke patients treated with MT [26]. They reported a poor correlation between CTP- predicted nonviable tissue and final infarct on DWI in stroke patients with periventricular hyperintensity Fazekas score 3 [26]. In view of the potential association between leukoaraiosis and cerebral hypoperfusion, a predefined and fixed relative CBF threshold value of < 30% could not accurately predict the final infarct. This could also help us to explain the relationship between moderate or severe leukoaraiosis and low spatial agreement that found in present study.

Besides that this was a retrospective study conducted in one stroke center, some other limitations should be also noted. First, the coregistration of basline CTP and follow-up DWI might be negatively influenced by different slice thicknesses. Second, besides DWI, some studies also evaluated the post-treatment infarct based on FLAIR images [9]. Meanwhile, the follow-up MRI was usually scanned at 3–7 days after MT in our study. Although not very significant, the post-treatment infarct area would potentially process within 7 days after MT. This wide spread would potentially affect the volumetric and spatial accuarcy. The optimal imaging modality and time points for assessing the follow-up infarct areas were still not determined. Third, only one automated perfusion analysis software was used to estimate the ischemic core. In view of the potential differences of volumetric parameters derived from different softwares [9, 14], further study comparing the spatial agreements among different perfusion analysis softwares was meaningful. Four, althoug Dice similarity coefficient was mostly used to assess the spatial accuracy, we must admit that it was strongly affected by false negative voxels especially in patients with small ischemic core on baseline CTP. Last, the ischemic core on baseline CTP was estimated based on a fixed threshold of CBF. Integrating multiple perfusion parameters using meachine learning had been reported to be useful in improving the accuracy of CTP in estimating the volume of ischemic core [27, 28]. Further study using mechine learning to combine the multiple perfusion metrics and clinical information for improving the spatial accuracy of CTP was anticipated.

## Conclusion

CTP could help to locate the post-treatment infarct on DWI with moderate spatial agreement in stroke patients who achieving successful recanalization after MT. Based on our results, we considered that the follow-up infarct could be effectively estimated based on the baseline CTP, especially when the volume of ischemic core was large and the leukoaraiosis was less severe. This result could help the clinicians to predict the outcome, and to establish the therapeutic strategy to a certain extent. Further study combining with the meachine learning method might be promising for improving the accuracy of CTP in quantifing and spatially locating the ischemic core in the patients with AIS.

## Data Availability

The data that support the findings of this study were available from the corresponding authors on reasonable request.
